# Safety analysis of the Weigao laparoscopic surgical robot for renal cyst decortication via transperitoneal and retroperitoneal approaches

**DOI:** 10.3389/fsurg.2026.1960246

**Published:** 2026-10-21

**Authors:** Run-Ze Pan, Hao Wang, Xin-Yu Liu, Xin-Lei Wang, Yu-Feng Sun, Bo Jiang, Zhi-Gang Li, Zhen-Duo Shi, Qing Liang

**Affiliations:** 1 Xuzhou Clinical College, Xuzhou Medical University, Xuzhou, Jiangsu, China; 2 Department of Urology, Xuzhou Central Hospital, Xuzhou, Jiangsu, China

**Keywords:** renal cyst decortication, retroperitoneal approach, robotics, transperitoneal approach, Weigao surgical robot system

## Abstract

**Objective:**

To evaluate the efficacy and conduct a safety analysis of the Chinese domestically produced Weigao Micro Hand laparoscopic surgical robot in renal cyst decortication via transperitoneal and retroperitoneal approaches.

**Methods:**

Clinical data were collected from 63 patients with renal cysts who underwent robot-assisted laparoscopic surgery between May 2023 and July 2025. Among them, 31 patients were treated via the transperitoneal approach and 32 patients were treated via the retroperitoneal approach. Clinical characteristics, as well as surgical and short-term postoperative outcomes, were prospectively collected and analyzed.

**Results:**

Both the transperitoneal and the retroperitoneal approaches were successfully completed without conversion to open surgery or other unplanned interventions. No statistically significant differences were observed between the two approaches in terms of the operative time (66.13 ± 24.99 min vs. 77.50 ± 27.62 min) or postoperative hospital stay (3.87 ± 1.48 days vs. 4.56 ± 1.08 days). The retroperitoneal approach was associated with significantly less intraoperative blood loss (15.68 ± 4.64 mL vs. 11.78 ± 3.80 mL, *P* < 0.05), whereas the transperitoneal approach was associated with a significantly shorter postoperative drainage duration (2.10 ± 0.98 days vs. 3.13 ± 1.62 days, *P* < 0.05).

**Conclusion:**

The application of the Weigao robotic surgical system in renal cyst decortication demonstrates favorable feasibility and safety. With respect to surgical approach selection, both techniques have their respective advantages and disadvantages. Given the continuity of laparoscopic experience, considerations for surgical safety, and the benefit of more refined manipulation afforded by the robotic system, the retroperitoneal approach currently appears to be the preferable option.

**Clinical Trial Registration:**

https://www.chictr.org.cn/showproj.html?proj=223823, identifier ChiCTR2400081949.

## 1 Introduction

Renal cysts are a common urological condition in clinical practice (1). They are classified into simple renal cysts and complex renal cysts. Simple renal cysts generally do not require treatment unless clinical symptoms develop, whereas complex renal cysts require follow-up or surgery to rule out malignancy. Simple renal cysts are common benign lesions, and indications for intervention include low back pain, hemorrhage, hydronephrosis, hypertension, and urinary tract infection. Currently, laparoscopic renal cyst decortication has become the mainstream treatment (2) because of its advantages of minimal trauma, rapid recovery, and a high cure rate. Compared with traditional laparoscopic surgery, surgical robots offer significant advantages such as better surgical field visualization, precise positioning, precise manipulation, tremor filtration, and the ability to overcome human physical limitations (3). As a result, they have been adopted by surgeons for various surgical procedures, with urological surgeons being among the leaders in their application (4).

In 2000, the da Vinci Surgical System became the first robot-assisted laparoscopic surgical system approved by the FDA for clinical use, and has since been widely applied both domestically and internationally (5). Currently, the da Vinci Surgical System has held a monopoly position in the field of medical robotics because of its superior performance and broad range of applications (6); however, its high equipment and maintenance costs limit its use in hospitals (7).

Since October 2021, when China's Weigao Micro Hand S robot passed the registration review of the National Medical Products Administration (NMPA) and was approved for the market, a number of Chinese surgical robots have emerged rapidly, and are receiving approval for clinical application (8). However, the practicality and generalizability of these emerging Chinese surgical robots still require further standardization and investigation across multiple levels of medical institutions, multiple specialties, and various surgical approaches (9). Larger-scale clinical trials, appropriate patient selection, and longer-term studies are needed (10) to ensure that cost-effective robotic surgery can benefit the broadest possible patient population.

This study represents the first prospective clinical investigation of the safety and usability of a laparoscopic surgical robot system for renal cyst decortication. We summarize the clinical outcomes of 63 completed robot-assisted laparoscopic renal cyst decortication procedures, as reported below.

## 2 Materials and methods

### 2.1 Study population

A prospective analysis was conducted on the medical records of 63 patients who underwent renal cyst decortication in the Department of Urology, Xuzhou Central Hospital, Jiangsu Province, China, from May 2023 to July 2025. All procedures were performed using the Weigao Micro Hand S laparoscopic surgical robot. This study is a prospective randomized controlled trial. A random number sequence was generated using SPSS software and placed in sealed, opaque envelopes. The surgical approach was communicated to the surgeon by a nurse who was not involved in the operation, immediately before the procedure.

Given that this is a single-center, exploratory study, and that high-quality prior literature directly comparing the retroperitoneal versus transperitoneal approach in terms of intraoperative blood loss for renal cyst decortication is relatively limited—with most previous studies reporting data as medians and ranges rather than as the means and standard deviations suitable for parametric estimation—we predetermined a sample size of approximately 30 patients per group (63 patients total) on the basis of clinical feasibility and the routine patient accrual period for similar procedures in our department.

### 2.2 Inclusion and exclusion criteria

The inclusion criteria were as follows: (1) Age 18–80 years; (2) Confirmed diagnosis of simple renal cysts by imaging examination, with a cyst diameter ≥4 cm and the presence of symptoms related tomass effect; (3) Preoperative evaluation confirming surgical indications and meeting the criteria for laparoscopic surgery; (4) No severe cardiopulmonary insufficiency or other major systemic diseases and ability to tolerate general anesthesia and surgery; and (5) Voluntary participation in the clinical study and consent to undergo robot-assisted laparoscopic surgery given by the subject or their legal representative/guardian.

The exclusion criteria for patients were as follows: (1) A preoperative imaging result suggesting a cyst diameter <4 cm; (2) A planned pregnancy, lactating, or pregnant during the entire clinical study period; (3) THE presence of severe cardiopulmonary disease, coagulation disorders, or other major systemic diseases that make the patient unable to tolerate surgery; (4) A history of gastric perforation, intestinal perforation, intestinal adhesion lysis, or prior surgery at the target surgical site; (5) The presence of other uncontrolled acute or chronic diseases that may affect postoperative recovery; (6) Concurrent abdominal infection, peritonitis, diaphragmatic hernia, or severe urinary or systemic infection; and (7) A refusal to undergo robot-assisted laparoscopic surgery.

This study was approved by the Medical Ethics Committee (approval no. XZXY-LK-20230427-066) and registered with the Chinese Clinical Trial Registry (registration no. ChiCTR2400081949). All patients provided written informed consent after being fully informed of the study.

### 2.3 Surgical equipment

All surgeries were performed using the Chinese domestically manufactured WeiGao Micro Hand S robotic surgical system. This system comprises a surgeon console, a patient console, and a 3D high-definition electronic vision system. Specifically, the surgeon console serves as the interactive platform for the operator and is positioned outside the sterile field. With the integrated display system, the surgeon sits at the console and views the operative field through a stereoscopic display while performing the procedure (Figure 1A). The patient console is the executing unit that facilitates minimally invasive surgery and is placed within the sterile field. It consists of a suspension arm, instrument arms, a base, and a column, and is connected to the surgeon console via dedicated cables (Figure 1B). The 3D high-definition electronic vision system provides real-time visualization of the laparoscopic surgical field during the operation.

**Figure 1:**
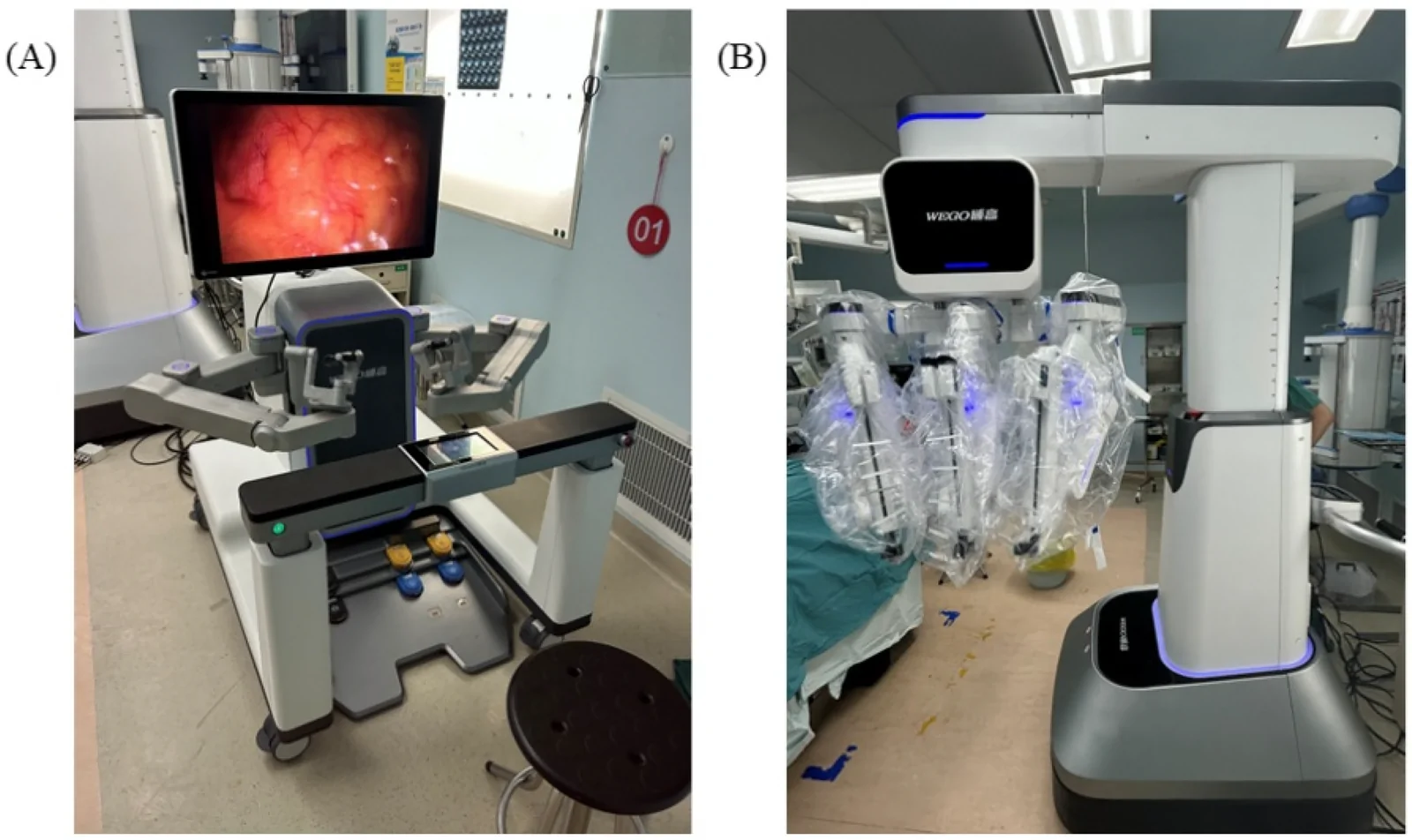
The weigao micro hands laparoscopic surgical robot. **(A)** Surgeon console; **(B)** Patient-side cart.

Laparoscopic surgical instruments included electrocautery, hemostatic forceps, laparoscopic scissors, and specimen retrieval bags. Robotic arm instruments include various direct-vision surgical tools, suction devices, hemostatic forceps, tissue cutters, and the like. Intraoperative monitoring equipment was used to continuously track the vital signs of the patients, including arterial blood pressure, real-time heart rate, and oxygen saturation.

All procedures were independently performed by experienced surgeons from the Department of Urology at Xuzhou Central Hospital. All the operating surgeons had completed standardized training on the WeiGao surgical robotic system and possessed extensive surgical experience.

### 2.4 Surgical procedures

All patients met the indications for robotic surgery. A detailed preoperative evaluation was performed for each patient, including medical history, physical examination, imaging studies (e.g., CT, MRI), and laboratory tests (e.g., complete blood count, urinalysis, electrolytes, renal function), to confirm surgical indications and exclude any contraindications.

Schematic diagram of surgical puncture sites via the retroperitoneal approach (Figure 2A) and the transperitoneal approach (Figure 2B).

**Figure 2:**
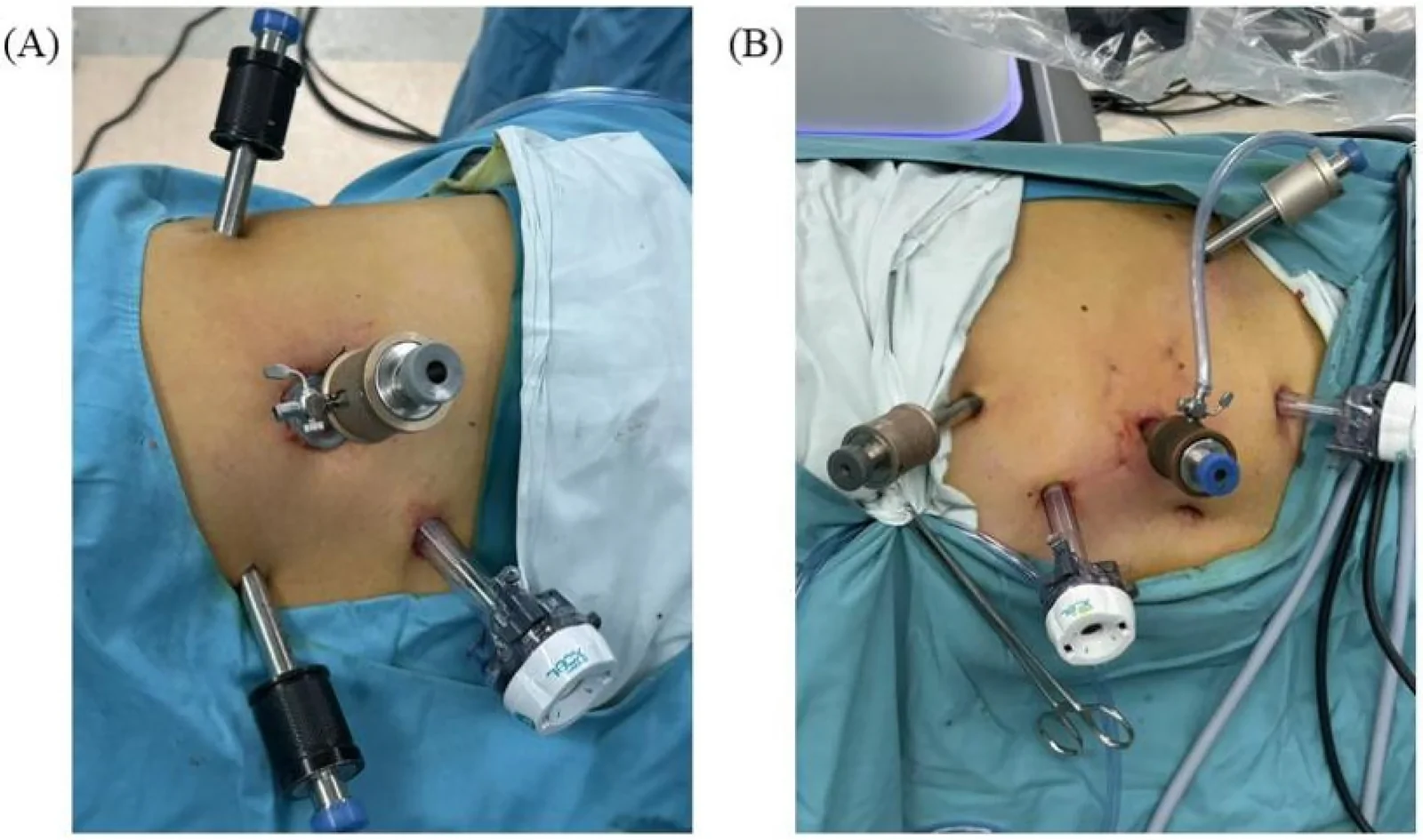
Trocar placement for robot-assisted laparoscopic deroofing of a renal cyst. (**A**)**:** Trocar placement for a left-sided retroperitoneal approach; (**B**)**:** Trocar placement for a left-sided transperitoneal approach.

#### 2.4.1 In the retroperitoneal approach

the retroperitoneal space is dissected, and Gerota's fascia is opened (Figure 3A). After the Gerota's fascia is dissected and exposed, the renal cyst is visualized (Figure 3B). The cyst fluid is aspirated (Figure 3C), after which the cyst wall is excised (Figure 3D). Hemostasis of the resection margin is achieved with an ultrasonic scalpel. After confirming that no active bleeding is occurring on the wound surface, a silicone drainage tube is placed in the perirenal space through a puncture site at the posterior axillary line, and each puncture site is sutured in layers.

**Figure 3:**
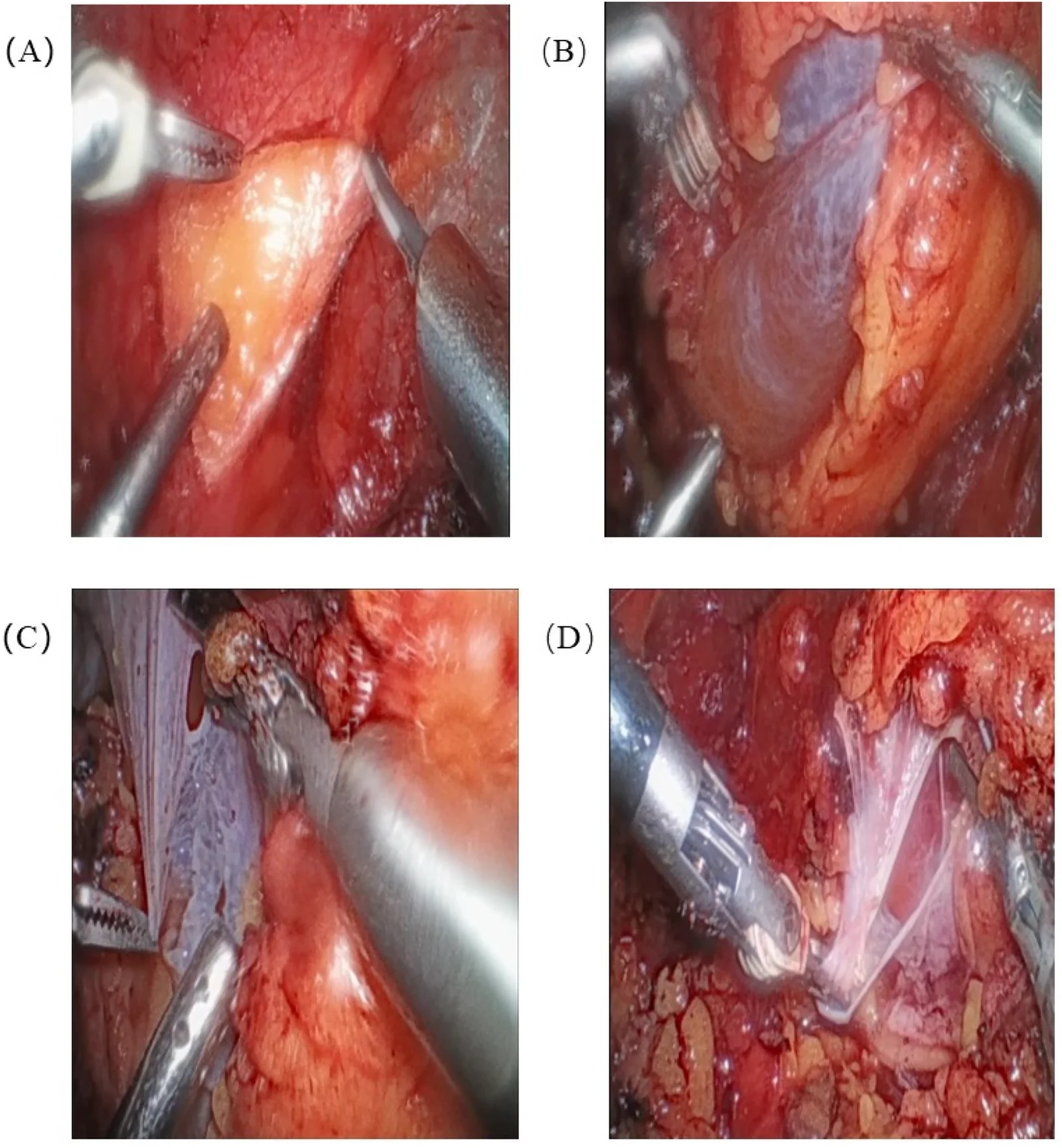
Surgical steps during robot-assisted laparoscopic deroofing of renal cyst. **(A)** Gerota's fascia opened; **(B)** Renal cyst exposed; **(C)** Cyst fluid aspirated; **(D)** Cyst wall excised.

#### 2.4.2 In the transperitoneal approach

the lateral peritoneum is incised along the line of Toldt, and the ascending colon is mobilized. The colon is mobilized and retracted medially. The triangular ligament, coronary ligament, and hepatocolic ligament are divided to separate the kidney from the liver and expose Gerota's fascia. Gerota's fascia is opened, and dissection is carried out with simultaneous electrocautery. The renal cyst is visualized, and after the cyst fluid is aspirated, the cyst wall is excised. Hemostasis of the resection margin is achieved with an ultrasonic scalpel.

### 2.5 Outcome measures

The following surgical-related parameters were collected and recorded for all patients: operative time, intraoperative blood loss, postoperative drainage duration, postoperative hospital stay, and the occurrence of postoperative complications.

### 2.6 Definitions of outcome measures

Successful surgery was defined as completion of the planned surgical objectives without conversion to conventional laparoscopic surgery or open surgery. Operative time was defined as the duration from skin incision to complete skin closure and was obtained from the surgical nursing records. Postoperative hospital stay was defined as the time from the day of surgery to discharge; for patients discharged in the morning, it was recorded as 0.5 days.

### 2.7 Statistical analysis

Statistical analyses were performed using SPSS 27.0. Continuous variables were first assessed for normality using the Shapiro–Wilk test, and the homogeneity of variance was evaluated on the basis of the variance ratio (largest variance/smallest variance). Since each group had a sample size greater than 30, the data were considered to approximate a normal distribution according to the central limit theorem; the variance ratios between groups for the primary and secondary outcomes were all far below 3 (intraoperative blood loss: 1.49; postoperative drainage duration: 2.73; length of hospital stay: 1.88), thus satisfying the assumption of homogeneity of variance for parametric tests. For the primary outcome (intraoperative blood loss), effect sizes (Cohen's d) and 95% CIs were calculated.

Continuous variables that met the assumptions of normality and homogeneity of variance are presented as the mea*n* ± standard deviation (*x¯* ± *s*), and between-group comparisons were performed using the independent samples *t*-test. Categorical variables are presented as counts (percentages) [n(%)], and between-group comparisons were performed using the chi-square test or Fisher's exact test (when expected frequencies were <5). All the statistical tests were two-sided, and a *P*-value <0.05 was considered to indicate statistical significance.

## 3 Results

### 3.1 Participant flow

A total of 78 patients with renal cysts were screened between May 2023 and July 2025. Among these patients, 11 were ineligible, and 4 declined to participate, resulting in 63 patients being randomly assigned to either the retroperitoneal approach group (*n* = 32) or the transperitoneal approach group (*n* = 31). All patients underwent the assigned robot-assisted cyst decortication without intraoperative crossover or conversion to open surgery. No patient was lost to follow-up. Therefore, all randomized patients (32 in the retroperitoneal group and 31 in the transperitoneal group) were included in the primary outcome analysis. The screening and randomization process is depicted in Figure 4.

**Figure 4:**
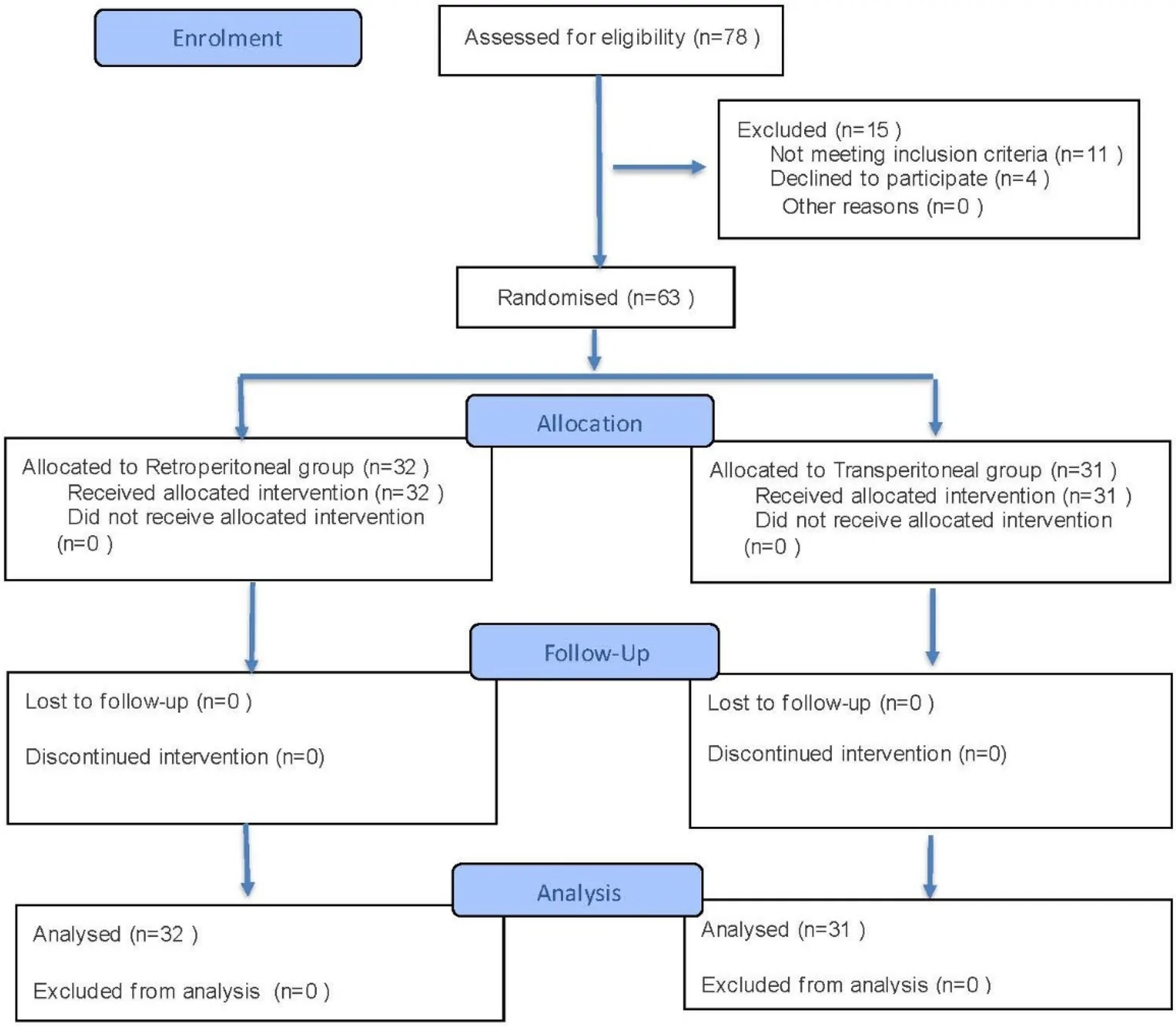
CONSORT 2025 flow diagram of the trial. Flow diagram of the progress through the phases of a randomised trial of two groups (that is, enrolment, intervention allocation, follow-up, and data analysis).

### 3.2 Comparison of clinical characteristics between the two groups

There were no statistically significant differences between the two groups in terms of age, sex, BMI, cyst location, or cyst size (all *P* > 0.05), as shown in Table 1.

**Table 1:** Comparison of clinical data between the two groups.

Variables	Transperitoneal group (*n* = 31)	Retroperitoneal group (*n* = 32)	*t/χ^2^/P* value
Age (years)	62.64 ± 7.98	59.34 ± 7.96	−1.642/0.106
Sex (male/female)	17/14	21/11	0.765/0.382
BMI（kg/㎡）	24.39 ± 2.89	24.80 ± 3.46	0.506/0.615
Cyst side (left/right)	17/14	16/16	0.148/0.701
Cystlocation (ventral/dorsal)	21/10	21/11	0.032/0.859
Maximum cyst diameter (cm)	6.62 ± 1.45	6.50 ± 1.22	−0.345/0.731

### 3.3 Comparison of perioperative parameters between the two groups

Comparisons of operative time and postoperative hospital stay between the two groups revealed no statistically significant differences (*P* > 0.05); however, statistically significant differences were observed in intraoperative blood loss and postoperative drainage duration (*P* < 0.05), as shown in Table 2.

**Table 2:** Comparison of perioperative period observation indices of the patients in the two groups.

Variables	Transperitoneal group (*n* = 31)	Retroperitoneal group (*n* = 32)	*t/χ^2^/P* value
Operative time (min)	66.13 ± 24.99	77.50 ± 27.62	1.712/0.092
Intraoperative blood loss (mL)	15.68 ± 4.64	11.78 ± 3.80	−3.654/＜0.001
Postoperative drainage duration (days)	2.10 ± 0.98	3.13 ± 1.62	3.61/0.002
Postoperative hospital stay (days)	3.87 ± 1.48	4.56 ± 1.08	1.778/0.08

### 3.4 Postoperative pathology and complications

In the transperitoneal approach group, one case (1.5%) of renal pelvis injury was reported. In this patient, intraoperative urine extravasation was noted. Postoperatively, management consisted solely of prolonged abdominal drainage (3 days) and fluid support; no invasive interventions, such as reoperation, interventional procedures, or retrograde endoscopic stenting, were performed. In accordance with the Clavien–Dindo classification (11), this complication was clearly graded as grade I.

In the retroperitoneal approach group, one case of postoperative recurrence was observed. At the 3-month post-operative ultrasound follow-up, a simple cyst (approximately 2.5 cm in diameter) was diagnosed, without any clinical symptoms related to renal cyst occupation, such as flank pain, lumbar distension, or hematuria. According to clinical consensus (12), simple cysts with a diameter <4 cm and without symptoms (Bosniak grade I) generally lack absolute indications for surgical or puncture sclerotherapy. Therefore, this patient did not require specific medical intervention at that time, and only regular imaging follow-up was recommended to monitor for any progressive enlargement of the cyst.

## 4 Discussion

Minimally invasive surgery, represented by laparoscopy, has become the preferred treatment modality in modern urology. With the advancement and development of medical technology, laparoscopic surgery is currently considered the optimal approach for renal cyst decortication in clinical practice (13). Among these, robot-assisted laparoscopic surgery is among the most promising and rapidly advancing surgical innovations of the 21st century (14). In recent years, intelligent surgical systems, such as the da Vinci surgical robot, have been widely applied in urology because of their significant overall efficacy (15). This system not only fully preserves the minimally invasive advantages of conventional laparoscopy (16) but also, through its high-precision robotic arm system and three-dimensional high-definition imaging technology, overcomes the limitations of traditional laparoscopy in terms of surgical flexibility and visual clarity (17). It enables surgeons to perform more delicate and complex procedures, ultimately achieving therapeutic outcomes comparable to those of open surgery (18, 19). However, the substantial costs, frequent maintenance, large system size, and need for appropriate surgeon training pose major challenges to further widespread adoption (20), which limits their promotion in prefecture-level city hospitals. In recent years, to reduce the application costs of surgical robots and facilitate their popularization, several domestically developed surgical robots (such as MicroPort's “Toumai” and Weigao's Micro Hand) have been successively introduced and put into clinical use.

In this study, 63 cases of renal cyst decortication were successfully completed with the assistance of the domestically produced Weigao Micro Hand S laparoscopic surgical robot, including 32 cases via the retroperitoneal approach and 31 via the transperitoneal approach. No perioperative complications of Clavien grade ≥Ⅱ were observed in either group. One case of renal pelvis injury with urine extravasation was reported in the transperitoneal group, and the patient was discharged uneventfully after symptomatic treatment. Intraoperatively, the robotic arms moved smoothly without any jamming or feedback delay. Limited visual field was encountered under extreme operating conditions, but this did not impede the overall fluency and precision of the procedure. Surgeons provided positive feedback on the high-definition visualization and image stabilization.

To date, no case-cohort studies on robot-assisted laparoscopic deroofing of renal cysts have been reported domestically or internationally. On the one hand, simple renal cysts are benign lesions, and laparoscopic deroofing is currently a well-established, safe surgical technique (13) that is widely regarded as an introductory procedure for laparoscopic urology. On the other hand, economic considerations are also important when a surgical approach is selected (21); robotic surgery is significantly more expensive than conventional laparoscopy in terms of operative costs (22). However, economic factors must be weighed against the safety and efficacy of the surgical procedure (23). A. Hinev et al. (13) reported outcomes of laparoscopic renal cyst decortication, with a mean operative time of 55 min (range, 30–95), mean intraoperative blood loss of 50 mL (range, 0–90), and a mean hospital stay of 4.2 days (range, 3–7). Compared with conventional laparoscopic surgery, our Weigao robotic system achieved a comparable operative time and hospital stay duration, with notably less blood loss, demonstrating its feasibility and safety for this procedure. Moreover, compared with a safety study of laparoscopic renal cyst decortication published in 2020 (24), our robot-assisted approach resulted in a significant reduction in postoperative complications, thereby substantially improving patient care quality (25, 26). This study preliminarily demonstrates that the application of the WeiGao robotic surgical system in renal cyst decortication is feasible and safe; however, further cases are still needed to support these findings.

Currently, two surgical approaches are available for the robot-assisted treatment of renal cysts: the retroperitoneal approach and the transperitoneal approach, each with its own advantages and disadvantages (27). In our study, the transperitoneal approach was associated with a shorter mean operative time (66.13 ± 24.99 vs. 77.50 ± 27.62 min), which can be attributed to the larger working space and clearer anatomical landmarks within the peritoneal cavity, facilitating surgical manipulation (27). Additionally, the transperitoneal approach yielded a shorter postoperative drainage duration (2.10 ± 0.98 days vs. 3.13 ± 1.62 days, *P* < 0.05), possibly because the retroperitoneal approach involves a deeper surgical bed, where fluid exudation tends to accumulate in the retroperitoneal space and is less easily drained, resulting in a one- to two-day delay in drain removal compared with the transperitoneal approach. However, the transperitoneal approach is more susceptible to interference from intra-abdominal organs (28), increasing the risk of visceral injury. In contrast, the retroperitoneal approach allows the robotic arms to directly access the perirenal space (29) without traversing abdominal viscera, thereby reducing interference with the bowel, omentum, and other tissues and lowering the risk of intraoperative bleeding and postoperative intestinal complications while more directly and safely exposing the kidney and its surrounding structures and minimizing mechanical injury to the kidney (30). Therefore, in terms of intraoperative blood loss, the transperitoneal approach was associated with greater blood loss than the retroperitoneal approach was (15.68 ± 4.64 vs. 11.78 ± 3.80 mL, *P* < 0.001). For the primary outcome measure (intraoperative blood loss), we calculated the effect size (Cohen's d) and its 95% confidence interval (CI). The results revealed an effect size of d = 0.92 (95% CI: 0.40–1.44) for the difference between the two groups. The lower limit of this CI (0.40) is well above the clinically recognized threshold for a small effect size (0.2) (31), and the entire interval lies within the moderate-to-large effect range, indicating that although the sample size was determined on the basis of feasibility, the observed effect of the retroperitoneal approach in reducing blood loss is statistically robust. However, caution is needed when these findings are interpreted. Although the effect size suggests statistical robustness, the absolute difference in intraoperative blood loss between the two groups was only 3.9 mL (95% CI: 1.81–5.99 mL), which is substantially lower than the conventional clinical threshold for blood transfusion and the routine measurement error range of the surgical suction canister (32, 33). This suggests that the advantage of the retroperitoneal approach in reducing blood loss may primarily reflect improvements in surgical precision, such as clearer anatomical planes or better control of capillary oozing, rather than yielding a substantive benefit with direct clinical decision-making value. Therefore, the clinical translation value of these findings is limited, and future studies should incorporate more accurate methods for measuring blood loss as well as post-operative recovery-related outcomes for further validation.

In the selection of a surgical approach for conventional laparoscopic renal cyst decortication, the transperitoneal approach is suitable for anterior, lateral, or larger renal cysts, whereas the retroperitoneal approach is more appropriate for posterior or dorsal cysts (e.g., those near the renal hilum or paravertebral) (27). However, tumor location is not the sole factor influencing surgeon choice (34). In robot-assisted laparoscopic surgery, on the one hand, owing to the continuity of long-accumulated laparoscopic techniques, and on the other hand, to minimize intraoperative injury and postoperative complications, surgeons often prefer the retroperitoneal approach (30). Xia et al. (35) reported that the retroperitoneal approach offers advantages over the transperitoneal approach in robot-assisted partial nephrectomy. More importantly, the flexible robotic arms, clear three-dimensional magnified imaging system, and ability to filter physiological hand tremors (36) enable surgeons to perform the majority of renal cyst decortication procedures via the retroperitoneal approach—a unique advantage of current robotic surgical systems.

This study has several limitations. First, this study had a single-center design with a relatively small sample size (*n* = 63), and the sample size was determined primarily on the basis of feasibility and the expected difference in the primary outcome measure (intraoperative blood loss), without *a priori* power calculation for rare complications or adverse events. Consequently, the ability to detect rare events such as Clavien–Dindo grade ≥ II complications is limited, and negative results should not be interpreted as definitive evidence of safety. Second, although all procedures were performed by surgeons with extensive experience in the retroperitoneal approach and the baseline characteristics were balanced between the two groups, we did not quantitatively assess the learning curve of the surgeons; differences in operative proficiency among surgeons may have influenced refined outcomes such as blood loss. Third, the follow-up duration was limited, precluding the capture of long-term complications and long-term functional recovery. Fourth, we compared only the retroperitoneal approach with the transperitoneal approach and did not include conventional multiport laparoscopic surgery as a comparator; thus, it is not possible to directly infer the superiority of the present approach over the current mainstream clinical technique. Fifth, we did not perform formal cost-effectiveness or cost-utility analyses; thus, the effects of surgical approach selection on healthcare resource utilization and patient economic burden remain unclear. In summary, the conclusions of this study warrant further validation through multicenter, large-sample, long-term follow-up randomized controlled trials that incorporate health economic evaluations.

Overall, robot-assisted laparoscopic deroofing of renal cysts represents a novel and effective surgical option. In terms of surgical approach selection, the retroperitoneal approach is currently the preferred choice.

## Data Availability

The original contributions presented in the study are included in the article/Supplementary Material, further inquiries can be directed to the corresponding authors.
